# A Very Rare Case of Predominantly Well-Differentiated Primary Male Breast Liposarcoma With Focal Dedifferentiation and Histologically Confirmed Pectoral Muscle Invasion

**DOI:** 10.7759/cureus.115950

**Published:** 2026-09-08

**Authors:** Muhammet A Yildiz, Duru C Gurbuz, Bahadır Öz, Mustafa Gok, Alper Akcan

**Affiliations:** 1 Department of Surgery, Erciyes University, Kayseri, TUR

**Keywords:** breast liposarcoma, core needle biopsy, male breast cancer, pectoral muscle invasion, well-differentiated liposarcoma

## Abstract

Primary breast liposarcoma is a rare subtype of breast sarcoma. In men, it is extremely rare and has generally been reported in the literature as case reports. An 82-year-old man presented with a painless, slowly growing mass in his left breast that had been present for 5-6 months. On examination, the mass measured approximately 20 cm and extended into the axilla. Mammography, breast MRI, and PET-CT demonstrated a fat-containing mass extending from the breast into the axilla. The mass had an indistinct border with the pectoral muscle, several solid areas, and mild FDG uptake (SUVmax, 2.5). Similar hypermetabolic areas were observed in the pectoral muscle and axilla. Core needle biopsy suggested dedifferentiated liposarcoma. The patient underwent simple mastectomy with resection of the pectoral muscle and axillary lymph node sampling. Final pathology of the surgical specimen showed a predominantly well-differentiated liposarcoma with two small foci of dedifferentiation and a low mitotic index, with no necrosis or pleomorphism identified, and confirmed histological infiltration of the pectoralis major muscle fibers. No metastasis was observed in the axillary lymph node sampling. The patient recovered well, and no adjuvant therapy was recommended. There was no recurrence or metastasis at 10 months of follow-up. This case demonstrates that pectoral muscle invasion, although rare, can occur in breast liposarcoma. It also demonstrates that the extent of dedifferentiation identified on biopsy may differ from that identified on final pathology. Finally, this case highlights that a definitive diagnosis requires comprehensive histopathological examination.

## Introduction

Primary breast sarcomas are rare, non-epithelial mesenchymal tumors derived from breast connective tissue and exhibit histological heterogeneity [1]. They account for less than 1% of malignant breast lesions, and liposarcoma is one of the rarest subtypes, accounting for less than 5% of breast sarcomas [2]. Liposarcoma exhibits a broad histological spectrum, ranging from well-differentiated liposarcoma (WDLPS)/atypical lipomatous tumor (ALT) to dedifferentiated liposarcoma [3]. Only a few published cases have described breast liposarcoma infiltrating the pectoral muscles [4]. To our knowledge, histological confirmation of true muscle-fiber infiltration has not previously been reported, as radiological or operative findings alone are insufficient to confirm true muscle-fiber infiltration. This gap between suspected and histologically confirmed muscle invasion may be related to the limited amount of breast tissue in men compared with women. Primary breast sarcoma is even rarer in men, with almost all available data derived from isolated case reports [5,6]. We report a very rare case of primary male breast liposarcoma with pectoral muscle invasion. The final diagnosis was predominantly well-differentiated liposarcoma with small areas of dedifferentiation. The closest previously reported example is that of Qiu et al. [4], who described a 68-year-old woman with a lipomatous tumor/well-differentiated liposarcoma of the breast adjacent to the chest wall muscle. Our case differs in that the patient was male, true skeletal muscle-fiber infiltration was confirmed histologically in the resection specimen, and the tumor exhibited a mixed-grade pattern with small foci of dedifferentiation that were not described in that report. Clinically, this distinction is important because it may directly affect prognosis and the decision regarding adjuvant therapy.

## Case presentation

An 82-year-old man presented with a five- to six-month history of a painless, slowly enlarging mass in his left breast. He had no family history, comorbidities, or history of substance use. On examination, a diffuse mass about 20 cm in size was palpated, filling the breast and axilla. It was non-tender, and there was no sign of skin involvement (Figure 1a). Mammography showed asymmetry in the left breast and axilla (Figure 1b). The nipple-areolar complex was normal, and no axillary lymph nodes were palpable. The contralateral breast, axilla, and supraclavicular fossa were all normal on examination. A breast MRI showed a mass extending from the left breast into the axilla, measuring about 20 x 10 cm, with an unclear border against the pectoral muscles. The mass was mostly fat, with several solid areas up to 2 cm, and it was reported as suggestive of liposarcoma (Figure 1c, 1d). PET-CT showed a fat-density mass measuring 116 x 67 x 125 mm confined to the breast component, with septations and solid components more visible on delayed images and mild hypermetabolic activity (SUVmax 2.5). Similar, mildly hypermetabolic areas were also seen in the pectoralis major and minor muscles and the left axillary fossa (Figure 1e, 1f). Core needle biopsy of the breast mass was performed, and pathology reported dedifferentiated liposarcoma. The patient underwent a simple left mastectomy with resection of the pectoralis major and minor muscles and axillary lymph node sampling (Figure 2a, 2b). The surgical wound was closed primarily (Figure 2c). Microscopic examination of the resection specimen revealed a predominantly WDLPS with two small foci of dedifferentiation (Figure 3a-3c). Histological examination of the resected pectoralis major and minor muscle margins confirmed direct infiltration of skeletal muscle fibers by the tumor, and all surgical margins, including the muscle margins, were free of tumor (R0 resection). Lymphovascular invasion was not seen. MDM2 fluorescence in situ hybridization (FISH) was attempted on the dedifferentiated foci; however, we observed hybridization failure in the targeted samples, precluding molecular confirmation. Immunohistochemistry was positive for vimentin and S100, supporting the mesenchymal, adipocytic nature of the tumor. He was discharged in satisfactory condition on postoperative day 5. Medical and radiation oncology did not recommend adjuvant therapy. At 10 months of follow-up, PET-CT and ultrasound showed no local recurrence or distant metastasis, a favorable short-term outcome that warrants continued surveillance rather than being interpreted as evidence of long-term prognosis. A summary of the clinical, imaging, operative, pathological, and follow-up findings is provided in Table 1.

**Figure 1:**
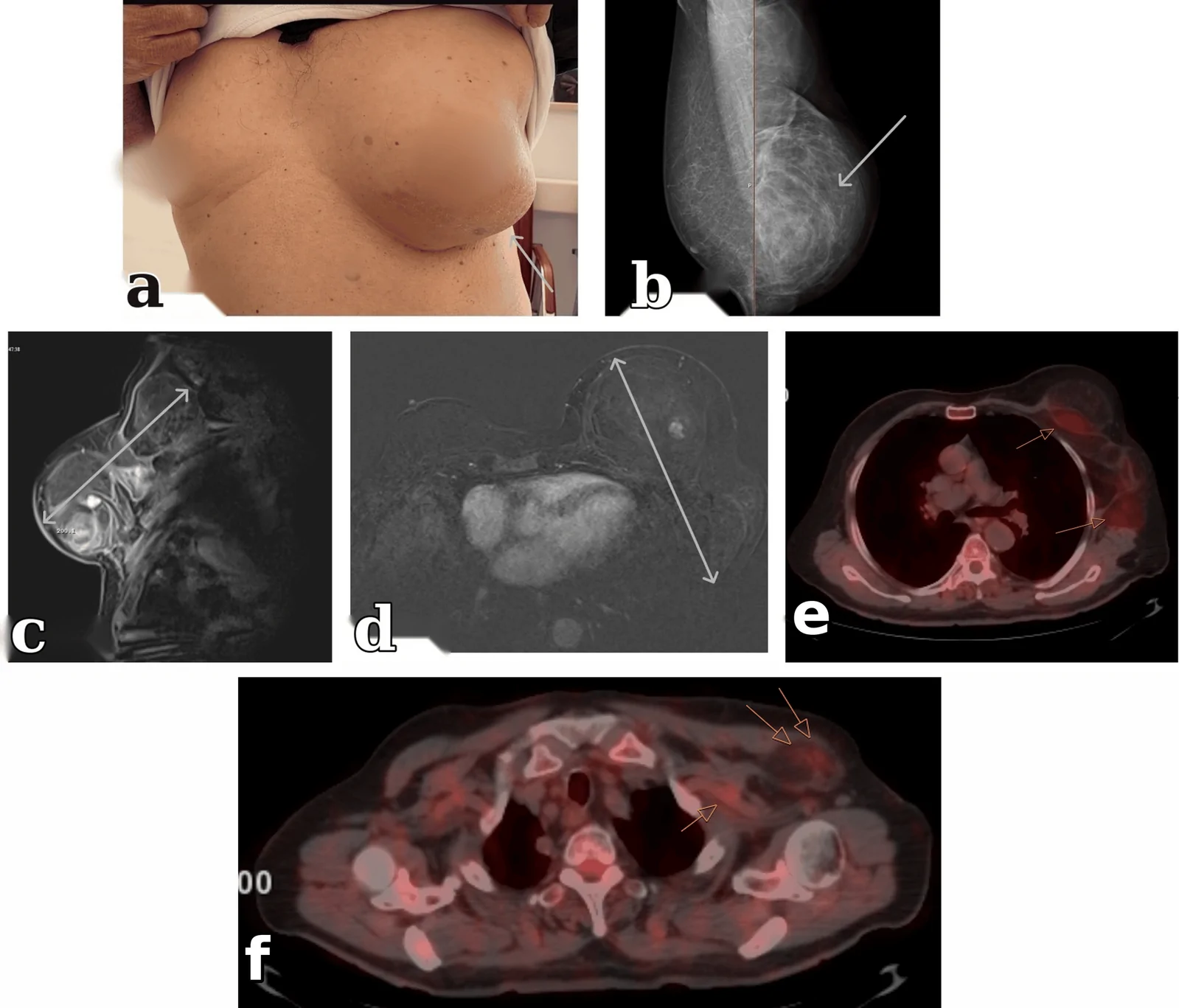
(a) Physical examination. (b) Mammography. (c, d) MRI. (e, f) PET-CT (a) Physical examination showing a diffuse mass filling the left breast and axilla. (b) Mammography showing asymmetry in the left breast and axilla. (c, d) Breast MRI showing a fat-predominant mass extending from the breast into the axilla (~20 × 10 cm), with an indistinct margin against the pectoral muscle and several solid components up to 2 cm, suggestive of liposarcoma. (e) PET-CT at the level of the breast/chest wall showing the fat-density mass with mild hypermetabolic activity; the orange arrows indicate the similarly mild hypermetabolic foci at the level of the pectoralis major muscle. (f) PET-CT at a more superior (axillary) level; the double orange arrow indicates the hypermetabolic focus at the level of the pectoralis minor muscle, and the single orange arrow indicates the hypermetabolic focus in the left axilla, read as suggestive of the primary tumor (liposarcoma) with possible metastatic foci, a radiological impression later reconsidered on final pathology.

**Figure 2:**
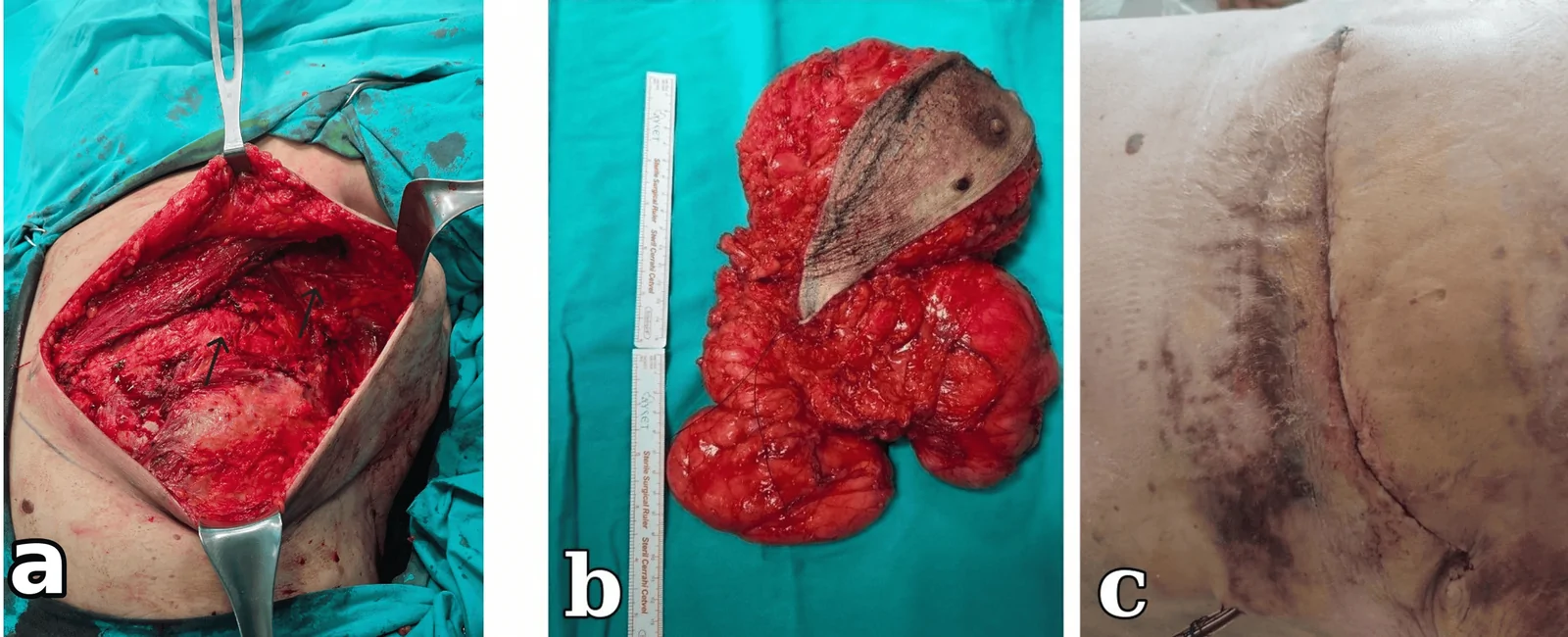
(a) Wide surgical exploration view during mastectomy, with black arrows indicating the resected portions of the pectoralis major and minor muscles. (b) Macroscopic view of the mass removed en bloc, including the skin, nipple-areola complex, and the adherent, invasion-suspected segments of the pectoralis major and minor muscles. (c) Postoperative view of the closed surgical incision

**Figure 3:**
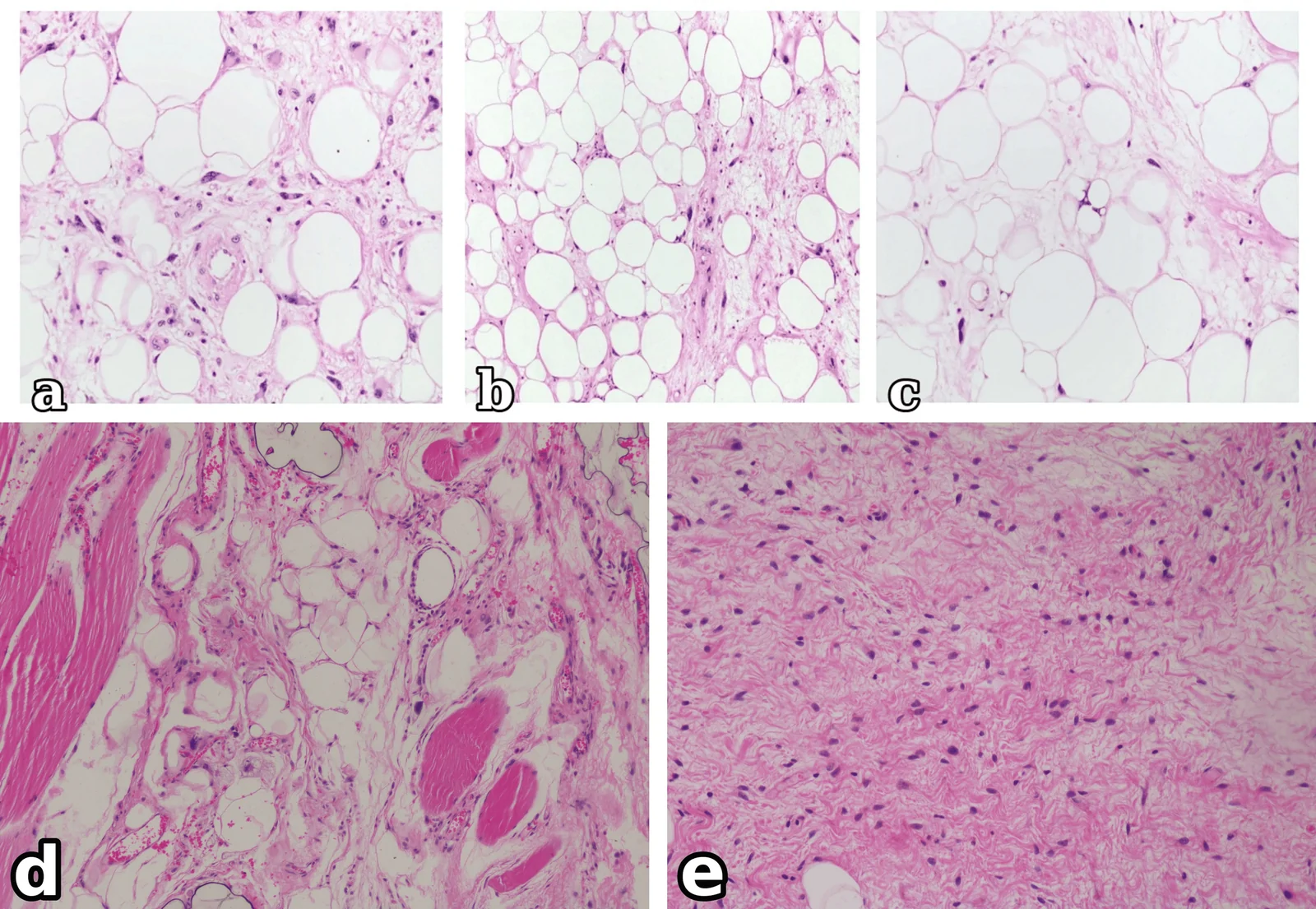
Microscopic view of specimen pathology (H&E stain, ×100) (a) Adipocytes of variable size separated by fibrous septa containing scattered atypical, hyperchromatic stromal cells, with a small vessel centrally. (b) Marked variation in adipocyte size (anisocytosis) with a thickened fibrous septum containing atypical spindle cells. (c) Fibrous septa with scattered hyperchromatic atypical cells and a small thin-walled vascular channel. (d) Tumor infiltration of skeletal muscle: atypical, hyperchromatic stromal cells and adipocytes of variable size are seen infiltrating directly between striated skeletal muscle fibers of the pectoral muscle. (e) Dedifferentiated focus: a densely cellular, non-lipogenic focus composed of a relatively uniform population of spindle-shaped, hyperchromatic stromal cells within a collagenous background.

**Table 1:** Summary of clinical, imaging, operative, pathological, and follow-up findings FISH: fluorescence in situ hybridization, IHC: immunohistochemistry.

Age/sex	82 years/male
Symptom duration	5-6 months
Mass size/location	~20 cm, left breast extending into the axilla
Mammography	Asymmetry, left breast/axilla
Breast MRI	~20 × 10 cm fat-predominant mass, indistinct margin against pectoral muscle, solid components up to 2 cm
PET-CT (primary mass)	116 × 67 × 125 mm, SUVmax 2.5
PET-CT: other regions (chest/abdomen/pelvis)	No additional uptake suggestive of distant metastasis
Core needle biopsy	Dedifferentiated liposarcoma
Operative procedure	Simple mastectomy + resection of invasion-suspected areas of pectoralis major and minor + axillary lymph node sampling
Final pathology: grade	Predominantly well-differentiated liposarcoma with two small foci of dedifferentiation
Dedifferentiated focus size/mitotic count/necrosis	Very small foci; low mitotic index (mitotic count 1-2 per 10 high-power fields); no necrosis or pleomorphism identified
Pectoral muscle invasion (histology)	Confirmed: tumor infiltrating skeletal muscle fibers
Lymphovascular invasion	No
Surgical margins	Clear/negative (R0)
Axillary lymph nodes sampled/status	Two suspicious nodes sampled; both reactive, no metastatic involvement
MDM2 FISH	Attempted on dedifferentiated foci; hybridization failed on FISH
Other IHC	Vimentin and S100-positive
Adjuvant therapy	Not recommended (predominantly well-differentiated grade, R0 margins, small dedifferentiated focus size)
Follow-up	10 months; PET-CT and ultrasound showed no local recurrence or distant metastasis: favorable short-term outcome

## Discussion

Liposarcoma is not a single disease but a spectrum. At one end of the spectrum is ALT/WDLPS; at the other is dedifferentiated liposarcoma, which arises from the same lineage but behaves a considerably more aggressive clinical course. Both share the same molecular hallmark: amplification of MDM2, usually together with CDK4, at chromosome 12q13-15. MDM2/CDK4 immunohistochemistry and fluorescence in situ hybridization (FISH), when necessary, can support the diagnosis when morphology alone is insufficient [7]. In our patient, the diagnosis was based primarily on histomorphological assessment of hematoxylin- and eosin-stained sections. MDM2 FISH was attempted on the dedifferentiated foci; however, the targeted samples failed to hybridize, precluding molecular confirmation. Dedifferentiated liposarcoma is aggressive and prone to metastasis, whereas ALT/WDLPS has a low recurrence rate and is relatively insensitive to radiotherapy and chemotherapy [3]. However, the two ends of the spectrum are not always clearly separated: a tumor can be predominantly well-differentiated but still contain a small dedifferentiated component, sometimes referred to as low-grade dedifferentiation, which carries a prognosis between that of pure ALT/WDLPS and fully dedifferentiated liposarcoma [8]. In our patient, the two dedifferentiated foci were focal, showed a low mitotic index, and had no necrosis or pleomorphism, consistent with the low-grade dedifferentiation pattern described by Lee et al. [8] rather than a higher-grade dedifferentiated component. Final pathology therefore reported the tumor as predominantly WDLPS with two small dedifferentiated foci, indicating that the case should not be considered a purely low-risk ALT/WDLPS.

Our case further highlights the difficulty of estimating the true degree of dedifferentiation in a small sample. Core needle biopsy suggested dedifferentiated liposarcoma, and the complete excision specimen confirmed dedifferentiation, but only as two small foci within an otherwise well-differentiated tumor. Needle biopsy is generally accurate for fatty tumors; however, its accuracy is reduced specifically in the ALT/WDLPS group, because a small sample from a large, heterogeneous tumor may result in over- or underestimation of the grade [9]. We did not directly correlate the biopsy site with the resection specimen. The breast quadrant from which the core biopsy was obtained was communicated to the pathology team, but the final report did not specifically confirm that the dedifferentiated foci corresponded to that site. Sampling error remains the most likely, although unconfirmed, explanation. The biopsy most likely captured one of these dedifferentiated foci, which, in a limited sample, could appear to represent a fully dedifferentiated tumor. Only examination of the entire specimen demonstrated that dedifferentiation was focal rather than diffuse. Although core biopsy can often determine tumor grade, comprehensive histological assessment of the resected specimen is required for an accurate diagnosis and appropriate treatment strategy.

Another notable feature of our case is the size of the mass at the time of diagnosis. Although it originated in the breast, it grew to more than 20 cm in total size and extended into the axilla. Reduced awareness that breast changes can also affect men is a recognized general barrier to earlier presentation of male breast disease, and in some reported cases a painless, slow-growing mass has remained unnoticed for an extended period [10]. However, our patient's relatively short 5-6-month symptom history suggests that the size of the mass at presentation is better explained by factors other than prolonged unawareness, most likely the tumor's growth rate. Other reported cases of male breast sarcoma likewise illustrate how such a mass can be mistaken for another condition, such as a phyllodes tumor or carcinoma, before the correct diagnosis is established [5,6].

Mammography and ultrasound usually yield nonspecific findings for such masses, and the fat content can sometimes be mistaken for a simple lipoma. MRI is the primary imaging modality used to evaluate lipomatous tumors, although distinguishing a lipoma from an ALT by MRI alone can still be challenging. On PET-CT, FDG uptake generally increases with tumor grade, with a stepwise rise in SUVmax from lipoma to ALT and then to myxoid and dedifferentiated liposarcoma [11]. The SUVmax of 2.5 in our primary mass is consistent with a predominantly well-differentiated tumor and is in keeping with the general increase in FDG uptake with increasing liposarcoma grade [11]. However, PET-CT cannot reliably exclude small dedifferentiated foci within a large, heterogeneous lesion. Our case illustrates this limitation, as two such foci were identified only on complete histopathological examination despite the overall low SUVmax. Thus, SUVmax should be regarded as supportive and correlative information rather than as a predictor of final pathological grade. The additional foci identified in the pectoral muscle and axilla on PET-CT were initially interpreted as metastases. The pectoral muscle focus specifically raised concern for direct tumor extension, whereas the axillary focus raised concern for axillary lymph node metastasis. Final pathology confirmed direct tumor infiltration of the pectoral muscle rather than a separate metastatic focus. The two sampled axillary lymph nodes were reactive, with no evidence of metastatic involvement. The final diagnosis was predominantly ALT/WDLPS rather than diffuse dedifferentiated liposarcoma. No progression was observed during 10 months of follow-up. Taken together, these findings confirm that the pectoral muscle focus represented direct tumor extension rather than metastasis, whereas the axillary FDG uptake most likely reflected reactive nodal or peritumoral changes associated with the large, fat-rich mass rather than nodal metastasis. Although imaging alone cannot entirely exclude metastasis from the small dedifferentiated component, follow-up has shown no evidence of metastatic progression. PET-CT foci suggestive of metastases should not be considered confirmed metastatic disease without tissue confirmation.

In our case, MRI and PET-CT could not clearly delineate the tumor border from the pectoral muscle. Pectoral muscle invasion is a rarely reported but recognizable feature of breast liposarcoma. In our patient, the radiological and operative suspicion of invasion was subsequently confirmed histologically in the resection specimen. The case reported by Qiu et al. closely resembles our findings and surgical approach. They described a 68-year-old woman with a breast lipomatous tumor/WDLPS with an indistinct margin from the chest wall muscle, which was managed with wide surgical excision [4]. In contrast, our patient was male, and histological examination of the resection specimen confirmed true skeletal muscle-fiber infiltration. In addition, our tumor showed two small foci of dedifferentiation, representing a mixed-grade pattern that was not described in the case reported by Qiu et al. These differences, rather than novelty alone, define the unusual features of the present case. Wide excision with negative margins remains the mainstay of treatment for breast liposarcomas. For ALT/WDLPS, the principal risk is local recurrence. Therefore, complete resection of any adjacent structure with suspected tumor invasion, such as the pectoral muscle in our case, is particularly important to achieve adequate local control.

The role of axillary lymph node dissection in breast sarcoma remains debated. In a national registry-based analysis of breast soft tissue sarcomas, lymphadenectomy was frequently performed despite the infrequent occurrence of nodal metastasis, and lymph node examination did not improve survival [12]. This finding is consistent with the broader principle that, unlike carcinomas, sarcomas typically spread hematogenously rather than through the lymphatic system; therefore, routine axillary lymph node dissection is not recommended. In our case, the hypermetabolic uptake observed in the axilla on PET-CT prompted lymph node sampling rather than complete axillary dissection. Both sampled nodes were subsequently reported as reactive, with no evidence of metastatic involvement, supporting this cautious approach.

Taken together, the biopsy-resection discordance and the limitations of PET-CT SUVmax discussed above illustrate the same principle: no single preoperative assessment method can reliably substitute for complete histopathological examination of the resection specimen when treatment decisions are being made. Surgery alone is often sufficient for pure ALT/WDLPS [3]; however, this evidence base applies most directly to tumors without a dedifferentiated component and was only one of several factors considered in our patient's management. When a tumor is predominantly well-differentiated but contains small dedifferentiated foci, as in our case, recurrence-free survival is generally worse than that of pure ALT/WDLPS but remains considerably better than that of fully dedifferentiated liposarcoma, with a reported distant metastasis rate of approximately 8% in this intermediate, low-grade dedifferentiated group [8]. The decision against adjuvant therapy was based on the absence of microscopic residual disease after wide excision, which achieved clear (R0) margins, including at the site of pectoral muscle invasion, together with the favorable histological features of the dedifferentiated foci described above. At the fully dedifferentiated end of the spectrum, the clinical course can be substantially more aggressive. In a case of breast dedifferentiated liposarcoma reported by Țigăran et al., multiple lung metastases developed only 12 months after radical mastectomy [13]. This contrast illustrates how the degree of dedifferentiation and the extent of tumor sampling can influence prognostic assessment and follow-up. The absence of recurrence or metastasis at 10 months represents a favorable short-term outcome but does not establish long-term cure, as even localized dedifferentiation warrants extended surveillance to determine whether this favorable course is sustained over time. We recommend clinical examination with breast/chest-wall imaging (PET-CT or ultrasound) every 3-6 months during the first two years.

## Conclusions

The prognosis of breast liposarcoma depends largely on histological grade and on the presence and extent of any dedifferentiated component. Purely well-differentiated tumors tend to do well, fully dedifferentiated tumors carry a much worse prognosis, and tumors with limited, focal dedifferentiation, as in our case, carry an intermediate prognosis. This grading cannot always be made reliably on core needle biopsy alone, so the diagnosis and the extent of any dedifferentiation should be confirmed by full examination of the resection specimen. Wide excision with negative margins remains the mainstay of treatment, and axillary lymph node dissection remains debated. A favorable early follow-up warrants cautious interpretation when dedifferentiation is focal.

## References

[1] Song B, Singh H (2024). Rare breast cancers review. Healthcare (Basel).

[2] Rubiera Valdés M, Gómez de la Riva I, Alonso de la Campa JM, Fernández-Velásco AA (2026). Pleomorphic liposarcoma of the breast. Rev Esp Patol.

[3] M S A, K C, Bhargavan RV, Somanathan T, Subhadradevi L (2024). An overview on liposarcoma subtypes: genetic alterations and recent advances in therapeutic strategies. J Mol Histol.

[4] Qiu G, Zhou X, Shang Z, Wang Y, Hai R (2025). Primary lipomatous tumor/well-differentiated liposarcoma of the breast with invasion of the pectoralis major muscle: a case report. Int J Surg Case Rep.

[5] Malik S, Ahluwalia C, Ahuja S (2024). A rare case of myxoid liposarcoma in the male breast masquerading as phyllodes tumor. Indian J Surg Oncol.

[6] Sucharita S, Sahu N, Giri R, Sahu SK (2023). Mammary not otherwise specified-type sarcoma with CD10 expression. J Cancer Res Ther.

[7] Nomura K, Tomita M, Kuroda K, Souda M, Chiba K, Yonekura A, Osaki M (2024). Overdiagnosis of atypical lipomatous tumors/well-differentiated liposarcomas by morphological diagnosis using only HE stained specimens: a case-control study with MDM2/CDK4 immunostaining and MDM2/CDK4 fluorescence in situ hybridization. BMC Cancer.

[8] Lee J, Moon S, Kwon HJ, Choe G, Lee KS (2025). Can dedifferentiated liposarcoma be reliably excluded based on mitotic rate? Prognostic and diagnostic implications of mitotic activity in liposarcoma. Virchows Arch.

[9] Thavikulwat AC, Wu JS, Chen X, Anderson ME, Ward A, Kung J (2021). Image-guided core needle biopsy of adipocytic tumors: diagnostic accuracy and concordance with final surgical pathology. AJR Am J Roentgenol.

[10] Iftikhar S, Burr E, Freigoun A (2026). Male breast cancer: growing insights and continuing challenges. Cancer Med.

[11] Oshiro H, Mizuta K, Tsuha Y, Aoki Y, Katsuki R, Tome Y, Nishida K (2024). Differential diagnosis of lipomatous tumors using (18)F-fluorodeoxygulcose positron emission tomography/computed tomography: a retrospective observational study. Cancer Diagn Progn.

[12] Gullett NP, Delman K, Folpe AL, Johnstone PA (2007). National surgical patterns of care: regional lymphadenectomy of breast sarcomas. Am J Clin Oncol.

[13] Țigăran AE, Abu-Baker A, Ion DE (2023). Extremely rare type of breast cancer-dedifferentiated breast liposarcoma-diagnosis and treatment. J Pers Med.

